# Controllable self-cleaning FET self-assembled RNA-cleaving DNAzyme based DNA nanotree for culture-free *Staphylococcus aureus* detection

**DOI:** 10.1186/s12951-024-02682-3

**Published:** 2024-07-15

**Authors:** Hui Wang, Ruipeng Chen, Yue He, Xiaoyan Zhu, Zhixue Yu, Zemeng Feng, Dongxia Pan, Liang Yang, Xiangfang Tang, Benhai Xiong

**Affiliations:** 1 State Key Laboratory of Animal Nutrition and Feeding, Institute of Animal Science, Chinese Academy of Agricultural Sciences, Beijing, 100193 P. R. China; 2 Institute of Subtropical Agriculture, Chinese Academy of Sciences, Changsha, 410125 China; 3 State Key Laboratory of Animal Nutrition and Feeding, College of Animal Science and Technology, China Agricultural University, Beijing, 100193 P. R. China

**Keywords:** Self-cleaning field effect transistor, DNA origami, Electrochemical biosensor, Carbon nanotube, Superhydrophobic-oleophobic coating

## Abstract

*Staphylococcus aureus* (SA) poses a serious risk to human and animal health, necessitating a low-cost and high-performance analytical platform for point-of-care diagnostics. Cellulose paper-based field-effect transistors (FETs) with RNA-cleaving DNAzymes (RCDs) can fulfill the low-cost requirements, however, its high hydrophilicity and lipophilicity hinder biochemical modification and result in low sensitivity, poor mechanical stability and poor fouling performance. Herein, we proposed a controllable self-cleaning FET to simplify biochemical modification and improve mechanical stability and antifouling performance. Then, we constructed an RCD-based DNA nanotree to significantly enhance the sensitivity for SA detection. For controllable self-cleaning FET, 1 H,1 H,2 H,2 H-perfluorodecyltrimethoxysilane based-polymeric nanoparticles were synthesized to decorate cellulose paper and whole carbon nanofilm wires. O_2_ plasma was applied to regulate to reduce fluorocarbon chain density, and then control the hydrophobic-oleophobic property in sensitive areas. Because negatively charged DNA affected the sensitivity of semiconducting FETs, three Y-shaped branches with low-cost were designed and applied to synthesize an RCD-based DNA-Nanotree based on similar DNA-origami technology, which further improved the sensitivity. The trunk of DNA-Nanotree was composed of RCD, and the canopy was self-assembled using multiple Y-shaped branches. The controllable self-cleaning FET biosensor was applied for SA detection without cultivation, which had a wide linear range from 1 to 10^5^ CFU/mL and could detect a low value of 1 CFU/mL.

**Supplementary Information:**

The online version contains supplementary material available at 10.1186/s12951-024-02682-3.

## Introduction

*Staphylococcus aureus* (SA), an anaerobic gram-positive bacterium, is widely distributed in natural environments and continues to pose a serious threat to human and animal health [1–3]. SA has strong adaptability and can tolerate a wide range of pH values, temperatures (7–48 °C, optimum 37 °C) and humidities [4]. In addition, SA can secrete more than 20 types of toxins and invasive enzymes [5], which may inhibit the host immune response, causing various infections, such as skin infections [6], pneumonia [7], osteomyelitis [8], and sepsis [9]. The World Health Organization (WHO) has classified SA as a high-priority health issue. To date, many new methods have been developed to detect SA [10], such as PCR [11], ELISA [12], aptamers [13], and CRISPR/Cas [14], which require relatively long detection times or complicated operations with a high probability of false-positive results. Therefore, a low-cost, simple-to-preparation, fast-detection, and convenient method is still needed.

Cellulose paper-based field-effect transistors [15] offer an affordable analytical platform for point-of-care diagnostics due to their inherent self-pumping ability, flexibility, low cost, degradability, and easy preparation [16–18], making them accessible even to consumers with limited budgets or no prior fabrication expertise. Numerous methods, including photolithography [19], vacuum filtration with metal stencils [20], inkjet printing [21], and wax printing [22], can construct hydrophilic and hydrophobic areas. However, due to the presence of hydrophilic hydroxyl groups, the hydrophilic area still easily absorbs water or organics, which results in poor mechanical properties and low sensitivity. In addition, the hydrophobic area cannot retain the three-dimensional pore structure, nor can it regulate the hydrophilicity and hydrophobicity on the surface.

The use of polymeric nanocomposites combined with O_2_ plasma treatment is a new method that can achieve controllable self-cleaning cellulose paper with different degrees of hydrophobic-oleophobic performance on the surface. Owing to the presence of short fluorocarbon chains, 1 H,1 H,2H,2H-perfluorodecyltrimethoxysilane (PTS) serving as a monomer has poor hydrophobic and oleophobic properties, but can polymerize to nano/microscale nanoparticles with relatively low surface energies. When polymerized PTS easily functionalizes with a hydroxyl group to form a rough surface structure, exhibiting strong hydrophobic-oleophobic properties. This approach offers a facile and cost-effective method for the fabrication of controllable self-cleaning cellulose paper or conductive wire, preventing the diffusion and adsorption of organic reagents. In addition, C-H bonds located in the long chain of PTS can be broken by O_2_ plasma, which effectively regulates the hydrophobic-oleophobic nature of the material surface. Therefore, polymerized PTS combined with O^2^ plasma treatment can regulate self-cleaning performance.

Single-walled carbon nanotubes (SWNTs) [23] are promising materials for use in both nanoelectronics and thin film devices, which can be used as a sensitive material but also as a conductive material depending on its chemical structure. Semiconducting SCNT (s-SWNT), which has mobility reaching 79,000 cm^2^V^-1^s^-1^ [24], is considered to be among the most promising sensitive materials for field-effect transistors. Metallic SWNT (m-SWNT) has a conductivity approaching 10^5^ S/cm with low resistance [25], and is suitable for application as conducting wires for field-effect transistors.

RNA-cleaving DNAzyme (RCD), which targets bacteria [26] is a short piece of DNA that can bind to another molecule with high specificity and use its catalytic activity to break the bonds between the nucleotides in the RNA. A new DNAzyme with a high affinity and specificity for SA was developed a simple lateral flow device with a high limit of detection (10^4^ CFU/mL) and long detection time (30 min) [27], which limits its use at the point-of-care. Therefore, there is an urgent need to establish an analytical device that c can inexpensively, quickly, reliably, and easily sense ultralow SA concentrations in complex biological samples.

DNA-origami [28, 29] is an effective technique for the precise construction of nanostructures through a bottom-up fabrication approach that has many advantages, such as relatively high yields, great robustness, and the ability to construct complex nonperiodic shapes. However, a long ssDNA (typically viral DNA ~ 7,000 nucleotides long) is needed as the main strand, and this DNA has high synthesis costs. For this reason, similar DNA-origami, which involves single or multiple strands of ssDNA specimens with varying lengths to hybridize and construct 1D to 3D nanoscale devices and structures based on the Watson Crick base complementary pairing principle, is proposed and defined by our group. Similar DNA-origami technology has the merits of traditional DNA origami technology. Moreover, this technology also has unique advantages, including low cost, simple design, and convenient synthesis.

**Scheme 1:**
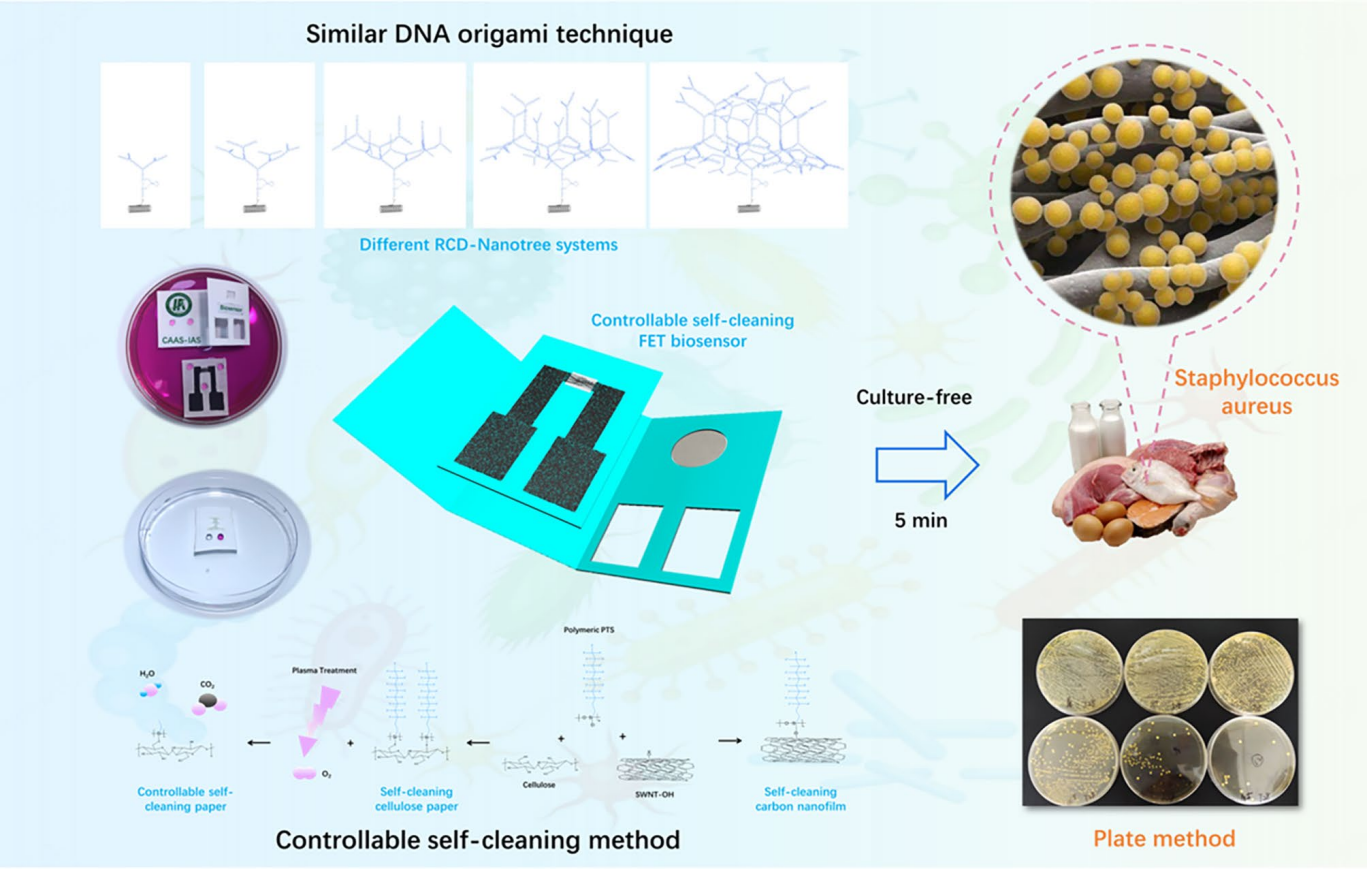
Schematically illustration of controllable self-cleaning FET self-assembled RNA-cleaving DNAzyme based DNA nanotree for culture-free staphylococcus au-reus detection

In this work, a controllable self-cleaning cellulose paper-based field-effect transistor was fabricated using cellulose paper as the substrate material, s-SWNT as the sensitive material and whole carbon nanofilm wire as the conductive electrode that was prepared using water-soluble conductive ink composited of SWNTs and hydroxypyrene. A polymeric PTS was synthesized and applied to improve the self-cleaning properties of cellulose paper and carbon nanofilm wire. The surface of the biosensing area was pretreated with O_2_ plasma to produce controllable self-cleaning cellulose paper. This step could maintain the hydrophobic and oleophobic properties inside the cellulose paper while improving the surface hydrophilicity, contributing to increase the number of SA-specific RCD modification on the surface of s-SWNT. Based on similar DNA-origami technology, different DNA structures were designed and hybridized with SA-specific RCD, which was applied to investigate the sensitivity. The detailed process is shown in Scheme 1.

## Materials and methods

### Chemicals and materials

Whatman filter paper (No. 1 with a pore size of 11 μm and No. 5 with a pore size of 2.5 μm) was purchased from Shanghai Jinpan Biotechnology Co., Ltd (China). Regular single-walled carbon nanotubes (SWNTs, purity ≥ 98%) were obtained from XFNANO (China). 1 H,1 H,2 H,2 H-perfluorodecyltrichlorosilane (PTS, 97%), 1-hydroxypyrene (HP, purity ≥ 98%), polydimethylsiloxane (PDMS) prepolymer (Sylgard 184 A) and the curing agent (Sylgard 184B) were bought from Sigma-Aldrich (China). Semiconducting single-walled carbon nanotube (s-SWNT, purity ≥ 90%) was obtained from Nano-Integris Inc (USA). 1-Pyrenebutanoic acid succinimidyl ester (PBASE) and bovine serum albumin (BSA) were obtained from Thermo Fisher Scientific (China). N, N-Dimethylformamide (DMF), n-hexane (purity > 95%), and ethanol (purity > 99.5%) were purchased from Macklin Biochemical Co., Ltd (Shanghai, China). Agar powder and LB broth could be obtained from Beijing Aoboxing Bio-Tech CO., LTD (China). Agarose (conventional), 1X TAE buffer, 10,000 × 4SGelred nucleic acid dye, and 6X glycerol gel loading buffer VII were provided by Sangon Biological Engineering Technology & Service Co. Ltd. (Shanghai, China). All chemical reagents were analytical grade and were used as received without further purification.

Three *Staphylococcus aureus* strains(BNCC 271,626, BNCC 326,053, BNCC 310,011) and the other strains including *Escherichia coli*, *Lactobacillus*, *Pasteurella*, and *Listeria* were purchased from the BeNa Culture Collection (Xinyang, Henan, China). All deoxyribonucleic acids in Table S1 and S2 were synthesized using standard solid phase techniques by a fully automated DNA synthesizer from Sangon Biological Engineering Technology & Service Co. Ltd. (Shanghai, China). According to the experimental requirements, the specific RCD for SA was designed with reference to the latest research findings [27], which had two chains, including SA-substrate and SA-DNAzyme. Branch-03 and Branch-05 with only one secondary structure shown in Figure S1 were further hybridized with SA-substrate and SA-DNAzyme through DNA triple helix structure.

### Apparatus

The surface morphology and energy dispersive spectroscopy results of the samples were analyzed using field-emission scanning electron microscopy (Hitachi, SU3500) at an accelerating voltage of 5 kV. The contact angle was recorded by an OCA 50 instrument (Dataphysics, Germany). Raman spectra were measured with Renishaw inVia instrument with an imaging microscope (532 nm diode and Ar ion lasers). Fourier transform infrared (FTIR) spectroscopy was performed on an FTIR spectrometer (Bruker Tensor-37, ranging from 540 to 4000 cm^− 1^ with a resolution of 0.5 cm^− 1^). A flexible electronics printer and plasma cleaning machine (CSCPIA305) were obtained from Shanghai Zhongpin Technology Co., Ltd. A vacuum pump was bought from LiChen Technology Co., Ltd(Beijing, China). The resistance and capacitance were measured by a digital multimeter (UT805A, China). Electrochemical data were collected by a CHI 760E electrochemical workstation (CHInstruments, China) at room temperature. The current-voltage (I-V) was measured by linear voltammetry, and the voltage was ranged from − 0.2 V to + 0.2 V (step + 0.01 V). The output characteristic curve of a field-effect transistor was obtained by a combination of the potentiostatic method (V_DS_=0.1 V) and linear voltammetry (V_G_ ranging from − 7 V to + 0 V). Blue LED Transilluminator (470 nm) and Electrophoresis Power Supply were purchased frome Sangon Biological Engineering Technology & Service Co. Ltd. (Shanghai, China).

### Bacterial culture and colony-forming unit (CFU) test

The frozen cultures of SA were activated with 5 ~ 10 mL Luria-Bertani (LB) broth and incubated for 12 h at 37 °C. A loop full of samples was taken from the growth of bacteria in the broth, and 0.2 mL of this loop was spread-plated on Man Rogosa Sharpe (MRS) agar and incubated for 24 h at 37 °C. This process was repeated three times to purify SA. Afterward, a 1 mm^2^ single colony was added into 5 mL LB broth and incubated for 4 h with continuous shaking at 37 °C and 200 rpm. The cultured SA was aliquoted into microcentrifuge tubes and stored at -20 °C until use.

The CFU count was the number of colony-forming units per milliliter of the original SA solution. One milliliter of the original SA solution was centrifuged at 1200 r/min for 5 min at room temperature to precipitate the SA. The supernatant was then removed. The precipitated SA was resuspended and diluted to an appropriate concentration. A total of 10 µL of diluted SA was inoculated and spread uniformly on a plate. The plate was then incubated at 37 °C for 24 h until visible colonies formed.

### Preparation of SC-FET/s-SWNT

**Fig. 1:**
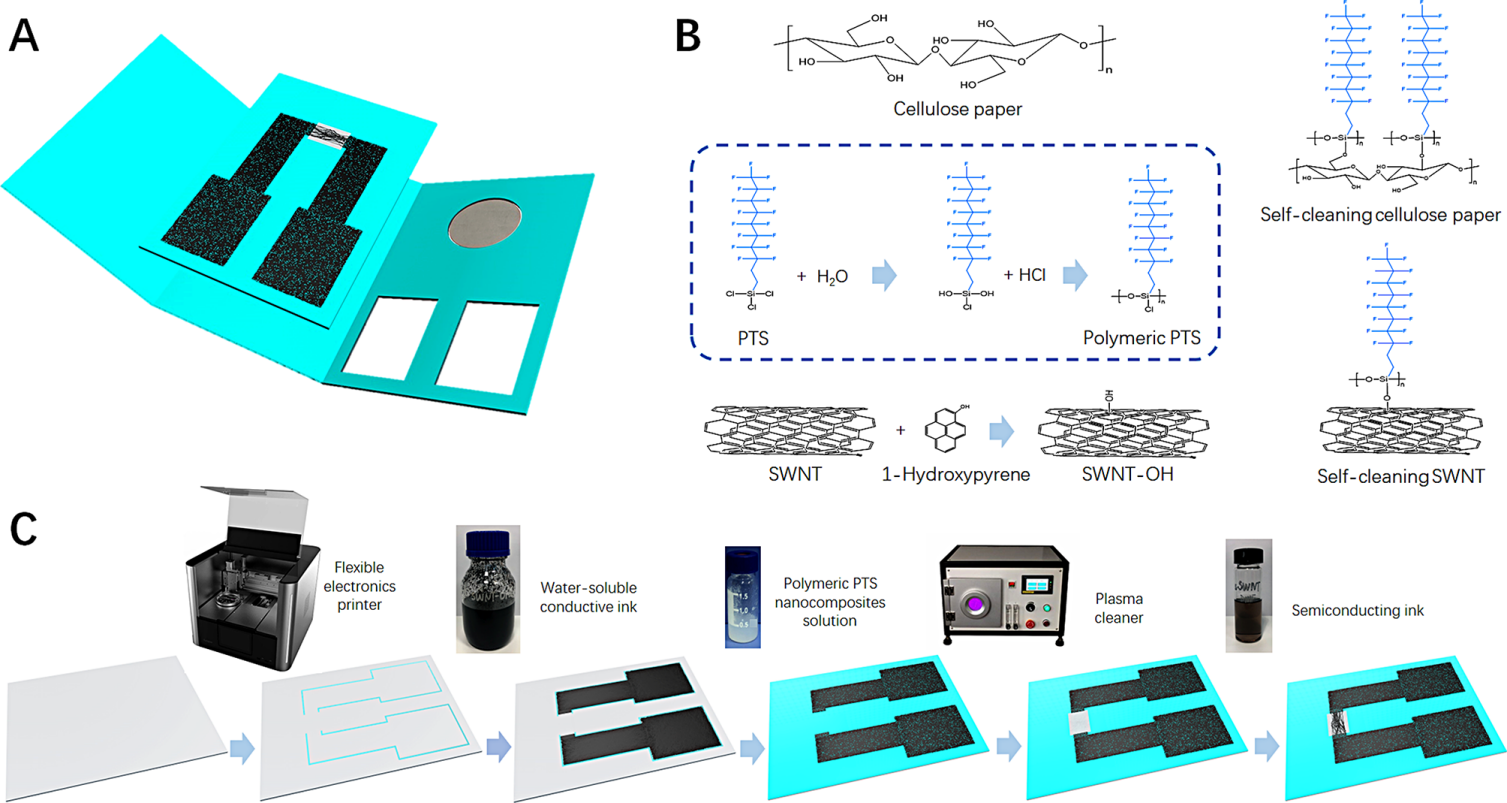
(**A**) Overall structure of the controllable self-cleaning cellulose paper-based field-effect transistor platform; (**B**) Synthesis mechanisms of polymeric PTS, SWNT-OH, self-cleaning cellulose paper, and self-cleaning SWNT; (**C**) Preparation of the self-cleaning field-effect transistor

Figure 1 shows the detailed preparation process of the controllable self-cleaning cellulose paper-based field-effect transistor platform. The overall structure shown in Fig. 1(A) had a filter-protective layer and field-effect transistor biosensor. Figure 1(B) shows the synthesis mechanisms of polymeric PTS that was functionalized with cellulose paper and hydroxylated SWNT to produce self-cleaning cellulose paper and SWNT.

#### Water-soluble conductive ink

A total of 50 mg of SWNT, 25 mg of 1-hydroxypyrene, and 100 mL of absolute ethanol were weighed and added into a glass bottle. The mixture was then sonicated, which is a process of applying high-frequency sound waves to a liquid, in an ice-water bath for 4 ~ 5 h until it was uniformly distributed. This process was performed to break up the SWNT into individual units, which were further combined with 1-Hydroxypyrene to dissolve in the ethanol. Finally, 200 mL of ultrapure water was added to the bottle to disperse the SWNT-OH solution evenly.

#### Polymeric PTS nanocomposites solution

A total of 0.4 mL of perfluorocarbon trichlorosilane and 20 mL of n-hexane were successively added to a glass bottle with a cap. Ultrapure water (40 µL) was mixed into the glass bottle and ultrasonically treated for 5 min, which allowed the water molecules to disperse uniformly with the mixing solution. After that, the glass bottle was slowly shaken until a light white suspension formed.

#### Semiconducting ink

First, 1.0 mg of s-SWNT and 50 mL of DMF were added to a glass bottle with a cap. The bottle was placed in an ice-water mixed solution via ultrasonic treatment for 4 ~ 5 h until a uniform, stable, and transparent solution was formed.

#### Self-cleaning field effect transistor

The pattern of field effect transistor structure in Fig. 1(C) was designed using AutoCAD software, and printed on Whatman No.5 cellulose filter paper by using a flexible electronics printer to create a PDMS outline. The PDMS-printed paper was then placed in a 90 °C oven for 10 min to consolidate the PDMS outline. Water-soluble conductive ink was first filtered on the source and drain electrodes under a vacuum force of 10.0 MPa and then washed with sufficient ethanol 3 times to remove residual 1-hydroxypyrene. The treated paper was immersed in a polymeric PTS nanocomposite solution with continuous shaking for 4 min, rinsed several times with sufficient n-hexane, and dried at room temperature, to prevent any organic or inorganic contamination. The biosensing element 4 mm in length and 2 mm in width was covered with a foil mask and treated with O_2_ plasma at 80% maximum power for 6 min, as shown in Figure S2. Based on the resistance changes, approximately 150 µL of semiconducting ink was dropped on the O_2_ plasma treated area and filtered by a vacuum force of 10.0 MPa, which was connected to the source and drain electrode.

#### Filter-protective layer

Whatman No.5 cellulose paper patterned with polymeric PTS nanocomposites was applied as the protective sleeve, and Whatman No.1 cellulose paper was cut and glued as a filtration membrane.

### Preparation of biosensors

#### Preparation of SC-FET/s-SWNT/RCD-Branch

##### SC-FET/s-SWNT/RCD-branch

The process of SC-FET/s-SWNT modified with different chemical and biological materials shows in Figure S2. PBASE (6 mM solution prepared in DMF) was non-covalently modified with s-SWNT through π–π bond formation between pyrene and s-SWNT, which was washed with sufficient ethanol by a vacuum force of 10.0 MPa. The PBASE-modified SC-FET/s-SWNT were incubated overnight with 20 µL of amine-labeled SA-substrate (50 µM) at 4°C for overnight. SA-substrate was immobilized covalently via an amide bond between the amine at the 5’ end and the ester groups of PBASE, and SA-DNAzyme was hybridized with SA-substrate to form SC-FET/s-SWNT/RCD. SC-FET/s-SWNT/RCD was incubated in 50 µM solutions of Branch-03 and Branch-05 for 2 h at room temperature, respectively, which was further covered with 0.1 mg/mL BSA solution for 1 h to block the excess ester group and naked s-SWNT. Finally, the prepared SC-FET/s-SWNT/RCD-Branch was immersed in pH 7.4 PBS solution at 4 °C for 48 h, which was used to remove possible residual DMF on the surface.

#### Preparation of SC-FET/s-SWNT/RCD-Nanotree

##### Y-shaped branch

Freeze-dried powders of three different ssDNA (YA-1, YA-2, and YA-3 in Table S2) were centrifuged at a speed of 10,000 rpm/min for 2 min, and then added sterilized PBS (pH 7.4) to prepare a concentration of 150 µM. Afterwards, equal volumes of YA-1, YA-2, and YA-3 were mixed together, heated to 95 °C for 3 min, and slowly cooled down room temperature to form YA, which was stored in a 4 °C refrigerator for future use. The branches of YB and YC were obtained using the same method described above.

##### SC-FET/s-SWNT/RCD-nanotree

SC-FET/s-SWNT/RCD fabricated by using the previous method in Sect. 2.5.1 was covered with 50 µM YA solution for 4 h, and then alternately hybridized with two complementary branches of YB and YC, with a hybridization time of 2 h each time. Different layers of SC-FET/s-SWNT/RCD-Nanotree were prepared based on the number of times, meanwhile, CS-B and CS-C ssDNA was applied to block the corresponding YB and YC. Afterward, the proposed biosensor was immersed in 0.1 mg/mL BSA solution.

### Sensing protocol

Due to insufficient preparation methods, it could be difficult to obtain electrochemical biosensors with completely consistent performance. Therefore, relative resistance was applied to reduce differences. Before use, the biosensor was stored in PBS with a pH of 7.4 at 4 °C. For SA detection, the original resistance was measured under optimal conditions. Afterward, the real sample was dropped on a filtration membrane for 5 min, and then the resistance was recorded again.

1 $${\text{Relative}}\,{\text{resistance}} = \frac{{{R_0} - R}}{{{R_0}}} \times 100\% $$

where ${R}_{0}$ and $R$ represent the resistance of biosensor before and after exposure to SA solution, respectively.

## Results and discussion

### Controllable self-cleaning performance of cellulose paper

**Fig. 2:**
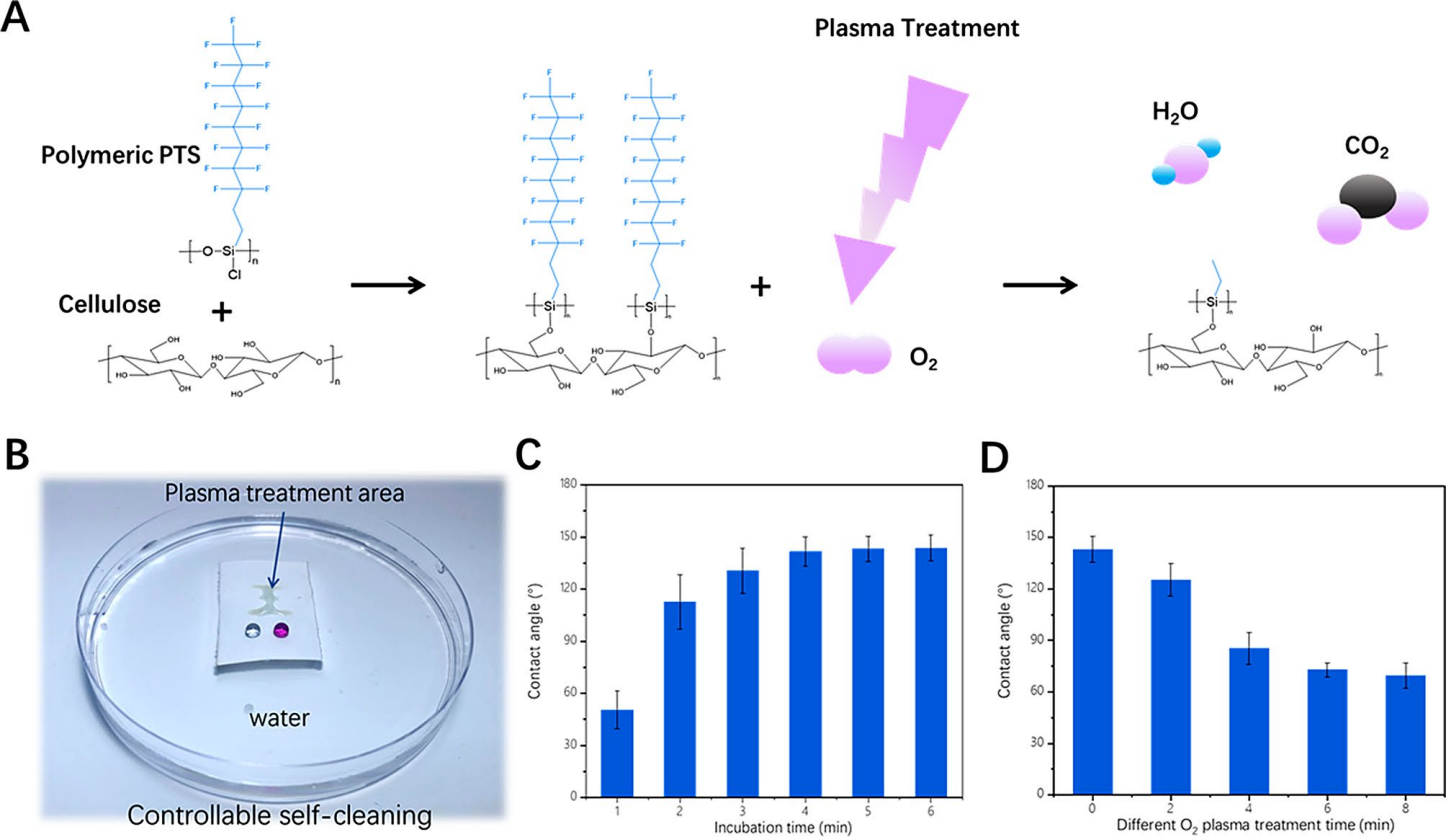
(**A**) Reaction mechanism of controllable self-cleaning cellulose paper; (**B**) Comparison of hydrophilic/hydrophobic properties between plasma-treated and untreated regions on self-cleaning cellulose paper; (**C**) Contact angle of self-cleaning cellulose paper affected by the incubation time of polymeric PTS; (**D**) Contact angle of self-cleaning cellulose paper changing with the O_2_ plasma treatment time

Figure 2(A) shows the reaction mechanism of controllable self-cleaning cellulose paper. This paper was produced by cellulose paper functionalized with polymeric PTS to form a self-cleaning cellulose paper, which was further treated through O_2_ plasma treatment to remove the fluorocarbon chain or polymeric PTS. Self-cleaning cellulose paper did not adherer to any flowing water in Figure S3(A), and different droplets of water, oil and organic reagent could maintained intact on the surface of elf-cleaning cellulose paper in Figure S3(B), demonstrating the hydrophobic-oleophobic property. After self-cleaning cellulose paper treated by O_2_ plasma, self-cleaning cellulose paper could clearly float well on the water surface, and only the O_2_ plasma treated area on its surface could combine well with the water to form a specific pattern in Fig. 2(B), indicating that O_2_ plasma treatment could control the hydrophobic-oleophobic property. Figure 2(C) shows the effect of incubation time between cellulose paper and polymeric PTS on self-cleaning ability, and Fig. 2(D) presents the effect of O_2_ plasma treatment time on the contact angle of self-cleaning paper, providing detailed parameters for controlling self-cleaning cellulose paper with different hydrophobic-oleophobic properties.

### Characteristics of SC-FET/s-SWNT

**Fig. 3:**
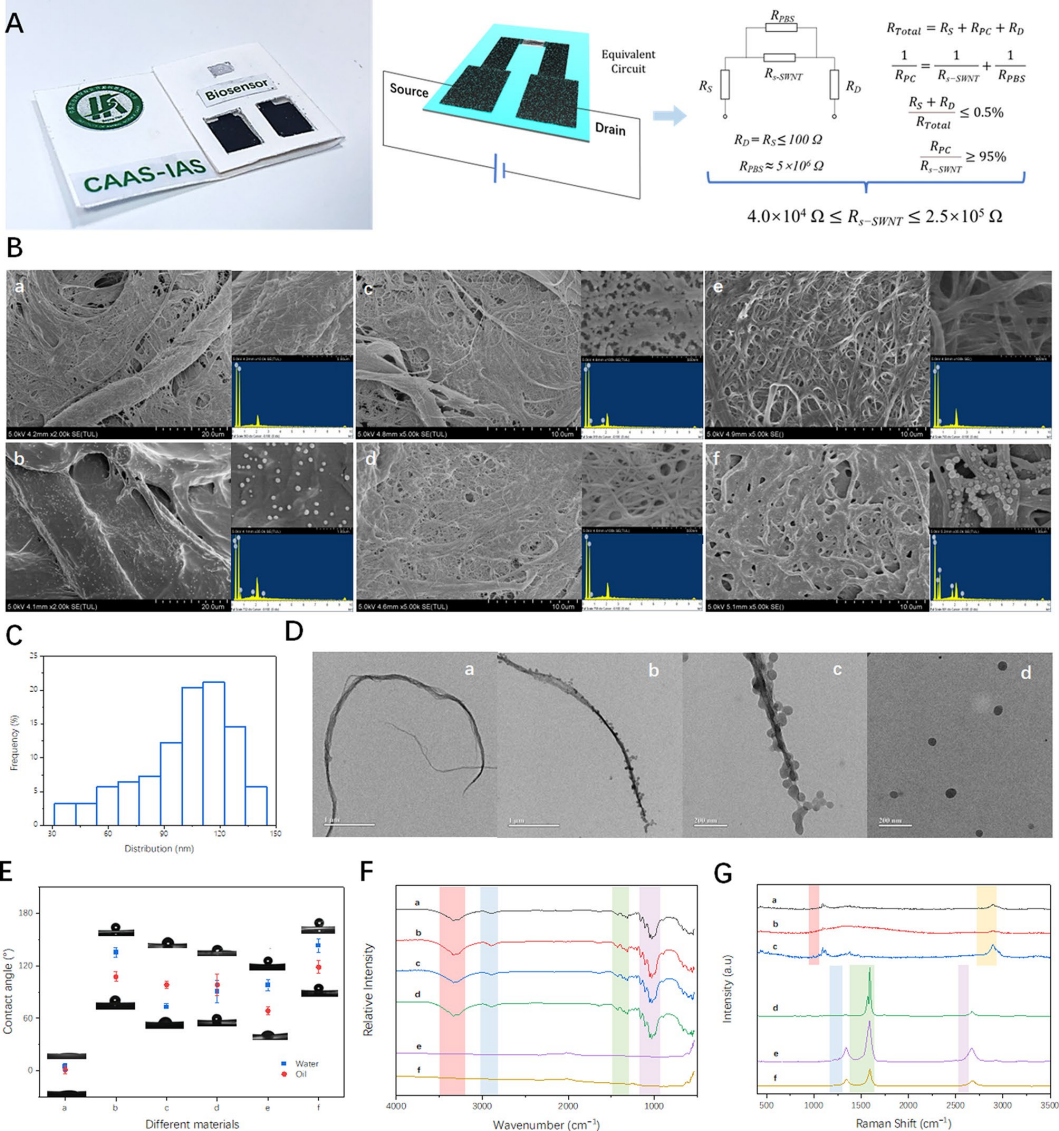
(**A**) Equivalent circuit and related calculation formulas of SC-FET/s-SWNT; (**B**) SEM and EDS of cellulose paper (**a**), polymeric PTS-cellulose paper (**b**), plasma-treated polymeric PTS-cellulose paper (**c**), s-SWNT immobilized on plasma-treated polymeric PTS-cellulose paper (**d**), SWNT-OH based carbon nanofilm (**e**) and SWNT-OH based carbon nano-film functionalized with polymeric PTS nanocomposites (**f**); (**C**) Distribution of polymeric PTS nanocomposites; (**D**) TEM of bare SWNT-OH (**a**), SWNT-OH/polymeric PTS nanocomposites (**b**); Enlarged image of SWNT-OH/Polymeric PTS nanocomposites (**c**) and Polymeric PTS nanocomposites (**d**); (**E**) Contact angle, (**F**) FTIR and (**G**) Raman of cellulose paper (**a**), polymeric PTS-cellulose paper (**b**), plasma-treated polymeric PTS-cellulose paper(**c**), s-SWNT immobilized on plasma-treated polymeric PTS-cellulose paper (**d**), SWNT-OH based carbon nanofilm (**e**) and SWNT-OH based carbon nano-film functionalized with polymeric PTS nanocomposites (**f**)

Figure 3(A) shows the carbon nanofilm-based SC-FET/s-SWNT, and the conductivity of carbon nanofilm was measured by using a four-point probe method. The surface resistance of the carbon nanofilm was approximately 21.45 ± 5.66 Ω/cm^2^, which was increased to 34.72 ± 4.24 Ω/cm^2^ after polymeric PTS nanocomposite decoration. The resistance of the source and drain electrode was less than or equal to 100 Ω. To reduce interference and improve sensitivity, an equivalent circuit of SC-FET/s-SWNT was constructed, where ${R}_{S}$, ${R}_{D}$,${R}_{s-SWNT}$ and ${R}_{PBS}$ represented the resistance values of the source electrode, drain electrode, semiconducting SWNT, and phosphate buffer solution. Based on the circuit calculation formula, the total resistance values of the series circuit and parallel resistance could be calculated. When the resistance of the source and drain electrode was lower than 0.5%, and the current of the semiconducting SWNT was much greater than 95% of that of the parallel circuit, we can conclude that the ${R}_{s-SWNT}$ ranged from 4.0 × 10^4^ Ω to 2.5 × 10^5^ Ω.

Figure 3(B) shows the scanning electron microscopy (SEM) image and energy dispersive spectrometer (EDS). The cellulose paper in Fig. 3(B-a) had a series of fiber bundles or streams, which were interwoven with many micropores for filtration. The diameter was typically within the range of a few micrometers, and the surface of the fiber paper was usually relatively smooth but could contain some small irregularities and particles. Upon closer inspection at high magnification levels, small fiber bundles and tiny pores were observed on the surface of the cellulose paper. Figure 3(B-b) clearly shows that an uneven semitransparent film and a large number of spherical nanoparticles were distributed on the fiber surface. Meanwhile, the fluorine content increased significantly, indicating that the polymeric PTS nanocomposites were uniformly functionalized on the cellulose paper. On the fiber surface of the plasma-treated polymeric PTS-cellulose paper, as shown in Fig. 3(B-c), there were numerous uniformly sized nanoscale pores and a relatively low fluorine content. When semiconducting SWNT was fixed on plasma-treated polymeric PTS-cellulose paper through suction filtration, a relatively sparse network structure was loaded on the surface with a high carbon-to-oxygen ratio, as shown in Fig. 3(B-d), especially on microporous surfaces. For the SWNT-OH based carbon nanofilm shown in Fig. 3(B-e), a dense network structure was present on the surface. After the polymeric PTS nanocomposites were modified, as shown in Fig. 3(B-f), many uniform and dense nanoparticles were distributed on the surface of SWNTs and the peaks of carbon and fluorine in EDS changed significantly in comparison with SWNTs-OH, illustrating good synergy.

**Fig. 4:**
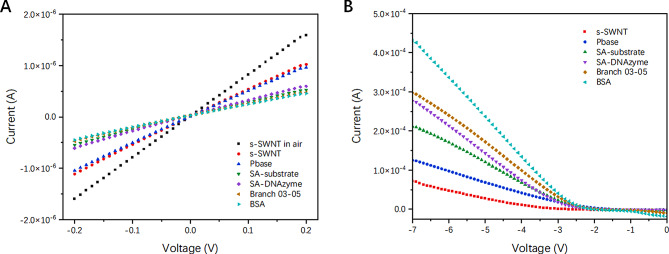
(**A**) Current-voltage characteristics and (**B**) Transfer curves of SC-FET/s-SWNT functionalized with Pbase, SA-substrate, DNAzyme, Branch-03, Branch-05 and BSA

Figure S4 shows the polymeric PTS nanocomposites evenly distribute on the surface of cellulose paper. Measurable maximum, minimum, and average sizes of polymeric PTS nanocomposites were 144.1 nm, 31.1 nm and 100.8 nm in Fig. 3(C), respectively. After statistics analysis, the particle size was mainly distributed in the range from 88 nm to 133 nm, accounting for approximately 68% of all PTS nanocomposites. Figure 3(D) show the TEM images of SWTN-OH before and after functionalized with polymeric PTS nanocomposites. Comparison with the bare SWNT-OH in Fig. 3(D-a), there were many polymeric PTS nanocomposites attached on the SWNT-OH surface in Fig. 3(D-b), which the partial enlarged image displayed in Fig. 3(D-c). It can be clearly seen that the PTS nanoparticles attached to SWNT-OH were relatively small, with an average particle size of approximately 51.2 nm. The detailed morphology characteristics of polymeric PTS nanocomposites are shown in Fig. 4Figure 3(E) shows the contact angles of cellulose paper before and after different treatments of water and oil were applied. The contact angles of the cellulose paper were almost zero for water and oil. The hydrophobic and oleophobic performance characteristics improved significantly after the polymeric PTS nanocomposites were used to treat cellulose paper that the contact angles reached 135.3°±4.53° and 107.67°±6.83° for water and oil, respectively. The main reasons for these angles were that the surface of the cellulose paper formed a micro/nanolevel structure and a hydrophobic molecular layer. The contact angles of plasma-treated polymeric PTS-cellulose paper were 72.88°±4.15° and 98.33°±5.63° for water and oil, respectively, suggesting that O_2_ plasma treatment only reduced the surface of polymeric PTS nanocomposites. After s-SWNT modification, the contact angles did not significantly change due to the low amount of s-SWNT. The contact angles of the carbon nanofilms were high, which contributed to the presence of hydroxyl groups on the SWNTs. The contact angles of the polymeric PTS nanocomposite-decorated carbon nanofilms reached 143.13°±6.37° and 118.66°±7.38°, respectively.

Figure 3(F) shows the FTIR spectra of the different functional groups and components of the cellulose paper before and after the different treatments. The typical peaks of cellulose paper showed absorption bands at 3341, 2911, 1428, 1319, and 671 cm^-1^, corresponding to O-H stretching vibration, C-H stretching vibrations, C-H bending vibrations, C-O stretching vibrations, and O-H bending vibrations, respectively [30]. When cellulose paper was functionalized with fluorinated silane polymers, the characteristic absorption peak of the Si-O-Si bond angular vibration group was identified at 574 cm^–1^, and the C–F and Si-O-C groups significantly improved the absorption peak within the range of 1250 ~ 1000 cm^-1^, suggesting the copolymerization of long-chain fluorinated amphiphilic with cellulose [31]. After O_2_ plasma treatment, the characteristic absorption peaks of the C–F and Si-O-C groups clearly decreased. The FTIR spectra of semiconductor single-walled carbon nanotubes usually did not have obvious structural characteristic peaks. Therefore, the following three curves exhibited little change.

Figure 3(G) shows the Raman spectra of cellulose paper before and after different treatments. Raman spectroscopy is a well-established technique used to investigate modifications of the electronic and vibrational properties of carbon nanostructures caused by interactions with different chemical species. The main peaks at 1094 and 2904 cm^-1^ in the spectrum of cellulose paper likely arose due to the vibrational modes involving C-O-C and C-H groups, which can be proved by the intensity change after cellulose paper was treated sequentially with polymeric PTS nanocomposites and O_2_ plasma sequentially. The Raman spectrum of SC-FET/s-SWNT exhibited four strong absorption bands located at 175 cm^-1^, 1342 cm^-1^, 1592 cm^-1^, and 1670 cm^-1^, which presented the radial breathing mode (RBM) region, the tangential mode (G-band) region [32]. After the composite of SWNT with hydroxy pyrene, two peaks of 1342 cm^-1^ and 1597 cm^-1^ were attributed to the D-band and G-band, respectively, because of the presence of defects and complete lattice vibrations in the SWNTs. The peak at 2671 cm^-1^ was attributed to the C-H bond vibration in hydroxy pyrene. The intensities of these characteristic peaks decreased after SWNT-OH was patterned with polymeric PTS nanocomposites, illustrating that the combination was good.

### Characteristics of SC-FET/s-SWNT/RCD-branch

To research the characteristics of SC-FET/s-SWNT functionalized with a DNA-nanostructure, SA-specific RCDs and two branches, as shown in Table S1 and Figure S1, were designed and synthesized to fabricate the SC-FET/s-SWNT/RCD-Branch. The preparation process in Figure S2 was described in the supporting information.

#### Electrochemical characteristics

Current-voltage (I-V) curves were measured using linear sweep voltammetry to characterize the electrochemical properties of SCFET/s-SWNT functionalized with different materials during fabrication. As shown in Fig. 4(A), I-V curves were observed over a range of − 0.2 V to + 0.2 V, which showed a good linear relationship, indicating that Ohmic contact sites formed between the s-SWNT-based sensitive area and the m-SWNT-based source and drain electrodes. When SCFET/s-SWNT was covered with pH 7.4 buffer solution, the currents decreased obviously because the OH^–^ ions can adsorb holes in s-SWNT. Pbase was modified to s-SWNT through π-π stacking interactions, but the current decreased slightly. This result arose because the electron-rich pyrene ring overlapped with the electron-deficient s-SWNT, resulting in a delocalization of electrons. After the SCFET/s-SWNT was immobilized with SA-substrate, SA-DNAzyme, four Branch and BSA, the currents at each voltage continued to decrease, except for SA-DNAzyme, which was attributed to the negative charge of both DNA and BSA (pl 4.7) in the pH 7.4 buffer solution. This means that the negatively charged DNA and BSA molecules occupied the number of holes in s-SWNT, which will further affect the conductivity of SCFET/s-SWNT. With respect to SA-DNAzyme modification, the current increased because the SA-DNAzyme bond with SA-substrate by base complementary hybridization decreased the entanglement of the DNA-negative backbone on the s-SWNT surface. The resistance values at -0.1 V were calculated using Ohm’s law, as shown in Figure S5.

The curve of a field-effect transistor (FET) is usually described by its output characteristics, which typically display the relationship between gate voltage (V_G_) and source-drain current (I_DS_). Figure 4(B) shows that when the source-drain voltage was held constant at 0.2 V with V_G_ sweping from − 7 V to 0 V, the I_DS_ linearly decreased between − 7 V and − 2.5 V. With chemical and biomaterial modification, the I_DS_ in the linear zone and gate capacitance between the s-SWNT and PBS solutions continuously increased, as indicated by the values listed in Table S3. According to the mobility equation, the mobilities of s-SWNT functionalized with Pbase, SA-substrate, SA-DNAzyme, Branch and BSA were 331.76, 274.01, 165.26, 190.19, 127.64, and 111.86 cm^2^/ V·s, respectively, which were in agreement with the I- V curves. The results remarkably demonstrate the positive hole of the p-type semiconductor SWNTs was occupied by the electron offered, which resulted in a reduced barrier concentration and carrier mobility.

#### Detection performance

Figure S6 shows the sensitive mechanism of SC-FET/s-SWNT/RCD-Branch for SA determination. The relative resistance was selected to reduce differences, but the voltage for measuring resistance had to be optimized. Figure S7(A) shows the current-voltage plots of SC-FET/s-SWNT/RCD-Branch used to detect the different SA concentrations in the range from − 0.2 V to 0.2 V with a scanning rate of 0.05 V/s. The current-voltage relationship was approximately linear, which could also be explained by the resistance values in Figure S7(B). Based on Formula 1, the relative resistance values for different SA concentrations were calculated at each voltage, as shown in Figure S7(C). Ignoring the values near 0 V, the relative resistance values of all SA concentrations were approximately equal at negative or positive voltages. To obtain the high sensitivity, -0.1 V was selected as the detection voltage in the experiment.

**Fig. 5:**
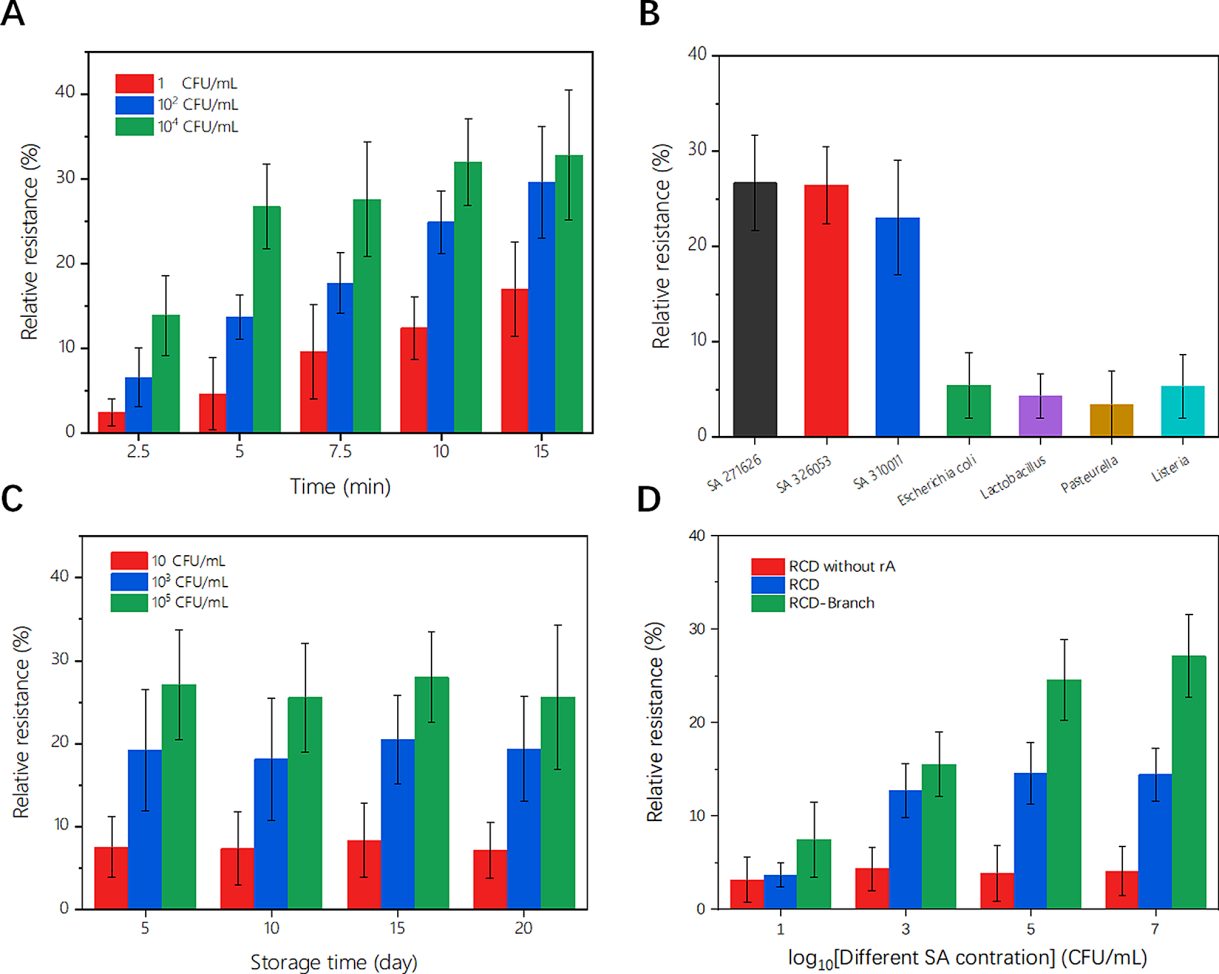
(**A**) Relative resistances of SC-FET/s-SWNT/RCD-Branch changing with different detection times for 1, 10^2^ and 10^4^ CFU/mL; (**B**) Selectivity investigations of SC-FET/s-SWNT/RCD-Branch for 10^3^ CFU/mL *Escherichia coli*, *Lactobacillus*, *Pasteurella* or *Listeria*; (**C**) Storage stability of SC-FET/s-SWNT/RCD-Branch at pH 7.4 and 4 ℃; and (**D**) Key factors affecting the sensitivity SC-FET/s-SWNT modified with RCD without ‘rA’ cleavage site, RCD and RCD-Branch

The detecting time is an important parameter for electrochemical biosensors. For SC-FET/s-SWNT/RCD-Branch, the detection time had two components: the incubation time and the measurement time, the measurement time (less than 10 s) could be ignored. Figure 5(A) shows the relative resistance values of SC-FET/s-SWNT/RCD-Branch exposed to 1, 10^2,^ and 10^4^ CFU/mL SA for different incubation times ranging from 0 min to 15 min. The relative resistance increased proportionally to the incubation time in the range from 0 to 5 min, and then the growth rate decreased significantly. Therefore, 5 min was chosen as the optimal incubation time.

To evaluate the selectivity, the interference characteristics of nonspecific bacteria were investigated. SC-FET/s-SWNT/RCD-Branch systems were immersed in different solutions containing 10^3^ CFU/mL *Escherichia coli*, *Lactobacillus*, *Pasteurella* or *Listeria*. The results, as shown in Fig. 5(B), revealed a clear distinction between the binding of SA and non-specific bacteria on the surface, thus confirming the selectivity of the developed SC-FET/s-SWNT/RCD-Branch system.

The storage stability was evaluated by using SC-FET/s-SWNT/RCD-Branch to determine three different SA (BNCC 271,626) solutions. Several proposed devices were fabricated and immersed in PBS (pH 7.4), which was then stored at 4 °C. Every five days, parts were removed and 10, 10^3,^ and 10^5^ CFU/mL SA solutions were added. By comparing the results in Fig. 5(C), there were no significant differences in the response of the devices over time, indicating that the proposed device had excellent stability.

Sensitivity is a vital parameter for electrochemical biosensors that directly affects detection accuracy. Figure 5(D) and Figure S8 show the relative resistance values of three biosensors with different RCD-based DNA structures. According to the red and blue bar charts, it indicates the cleavage site of SA binding to RCD was located at ‘rA’, indicating the change in the RCD structure on the s-SWNT surface. After SC-FET/s-SWNT/RCD was modified with Branch-03 and Branch-05, the relative resistance increased significantly at the same SA concentration. These phenomena arose mainly due to the negative charge of the DNA phosphate backbone, which occupied the holes in the s-SWNT to affect the conductivity. It was also proven that similar DNA-origami technology is an effective method for improving the sensitivity of controllable self-cleaning FETs. The linear relationship of SC-FET/s-SWNT/RCD-Branch for three SA was shown in Figure S9. In addition, when SC-FET/s-SWNT/RCD-Branch was applied to measure the SA, the RCD-Branch was broken at ‘rA’ that cannot be reconstructed. All above reasons indicate that SC-FET/s-SWNT/RCD-Branch had poor reusability that cannot be used multiple measurements. Therefore, the reusability not discussed in the article.

### Sensitivity enhancement by designing synthetic RCD-nanotree

**Fig. 6:**
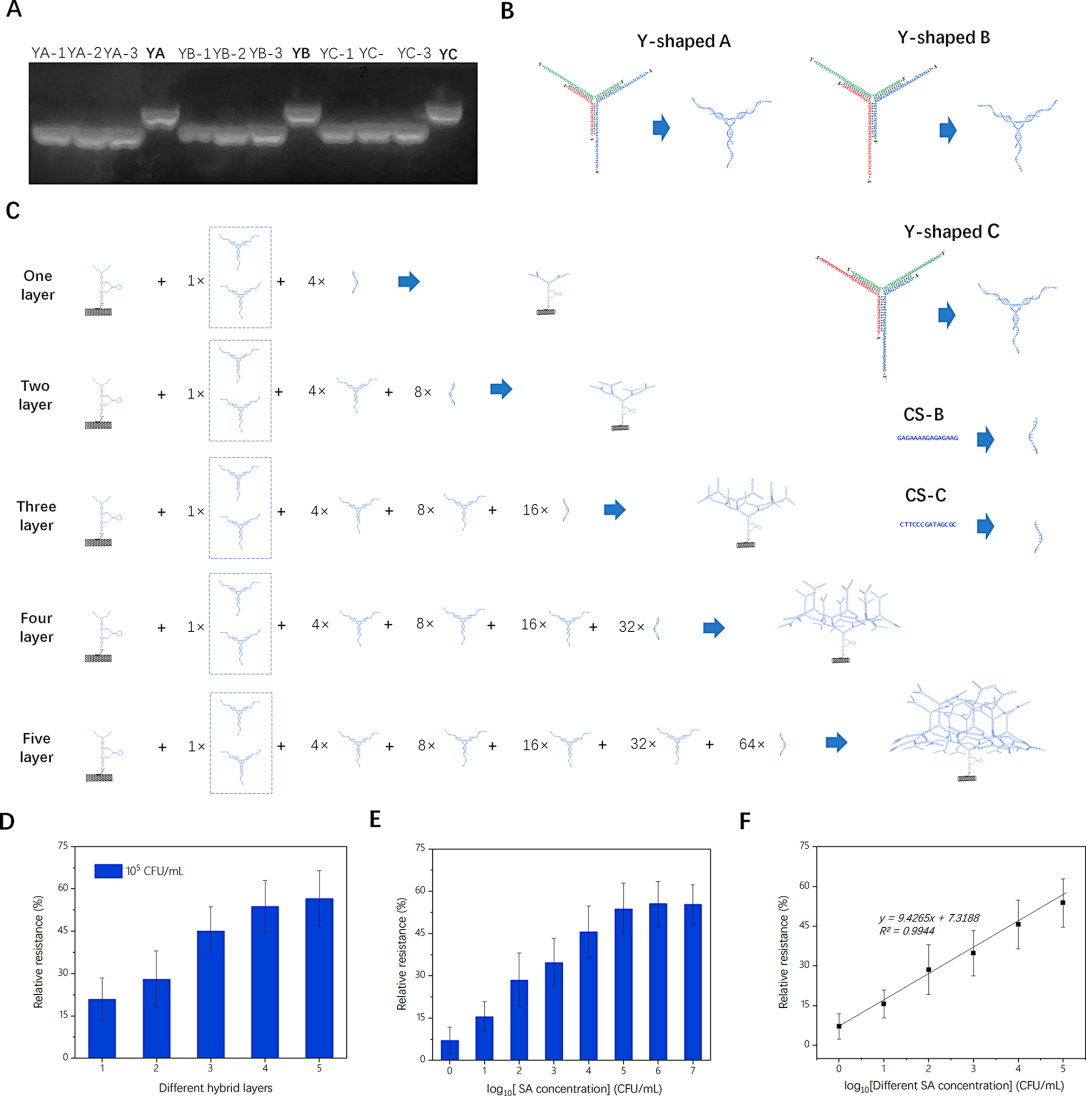
(**A**) Electropherograms of different single-stranded DNAs and self-assembled Y-shaped branches; (**B**) Sequence structure of three different Y-shaped branches and CS DNA; (**C**) SC-FET/s-SWNT/RCD self-assembled with different Y-shaped branches and CS DNA to construct SC-FET/s-SWNT/RCD-Nanotree systems ; (**D**) Comparison of relative resistances of five different SC-FET/s-SWNT/RCD-Nanotree for 10^5^ CFU/mL; (**E**) Relative resistances of the optimal SC-FET/s-SWNT/RCD-Nanotree systems after exposure to different SA concentrations(10^0^, 10^1^, 10^2^, 10^3^, 10^4^, 10^5^, 10^6^, and 10^7^ CFU/mL); (**F**) Linear regression curve for the detection of different SA concentrations ranging from 1 to 10^5^ CFU/mL

Based on the aforementioned research findings, the sensitivity of biosensors could be significantly enhanced by incorporating additional negative DNA nanostructures on the surface of SC-FET/s-SWNT. Due to the high cost associated with synthesizing commercial single-stranded DNA sequences larger than 150 nucleotides, similar DNA-origami technology was proposed to develop complex DNA nanostructures. Therefore, three different Y-shaped Branches were redesigned, and assembled with RCD in a layer-by-layer manner. Table S2 shows the corresponding DNA sequences. The electrophoretic patterns in Fig. 6 (A) proved that stable Y-shaped DNA structures could be formed through three complementary single-stranded DNA strands. Figure 6(B) shows three different Y-shaped DNA structures and two complementary DNA strands. By employing a layer-by-layer self-assembly hybridization approach, the controllable construction of DNA nanostructure could be achieved through two complementary Y-shaped branches. Figure 6(C) shows the detailed hybridization of five different SC-FET/s-SWNT/RCD-Nanotree, in which each step was maintained at room temperature for 2 h. Then, five SC-FET/s-SWNT/RCD-Nanotree systems were applied to evaluate the sensitivity by measuring 10^5^ CFU/mL SA, and the results are shown in Fig. 6(D). By balancing preparation complexity, cost, and sensitivity, it was found that FET/s-SWNT/RCD-Nanotree with four layers could achieve optimal sensitivity.

As shown in Fig. 6(E), four layers of SC-FET/s-SWNT/RCD-Nanotree were chosen to detect the different SA concentrations ranging from 1 to 10^7^ CFU/mL. The relative resistance values showed dynamic variations with the logarithm of the SA concentration in the range from 1 to 10^5^ CFU/mL. The linear regression equation was $$Relative\text{ }resistance=9.4265{log}_{10}\left(\text{S}\text{A} \text{ }\text{c}\text{o}\text{n}\text{c}\text{e}\text{n}\text{t}\text{r}\text{a}\text{t}\text{i}\text{o}\text{n}\right)$$$$+7.3188 ({R}^{2}=0.9944)$$ with a detection limit of 1 CFU/mL. Table 1 presents a comparison of the proposed biosensor with previous reports. The detection of SA using SC-FET/s-SWNT/RCD-Nanotree could be achieved in two simple steps without complex operations. Additionally, the proposed device had a low LOD and short detection time. Although the linear range was not the widest, it was very suitable for detecting ultralow SA concentrations. Therefore, the overall performance of the SC-FET/s-SWNT/RCD-Nanotree composite was better than that in previously published works.

**Table 1:** Comparison of the proposed biosensor with previous reports

Biosensor	Method	Operational complexity	Time (min)	Linear range (CFU/mL)	LOD (CFU/mL)	References
Electrochemical biosensor based on atriple-helix molecular switch	DPV	High	> 30	30 ~ 3 × 10^8^	8	[33]
CRISPR/Cas12a based fluorescence-enhanced lateral flow biosensor	Colour/ Fluorescence	High	70	-	5.4 × 10^2^	[34]
Gold nanoparticle electrode	SWV	High	1	10 ~ 10^7^	3	[35]
Aptamer electrochemiluminescence biosensor	ECL	High	50	10 ~ 10^7^	3	[36]
Ultrasensitive electrochemical aptasensor using tyramide-assisted enzyme multiplication	Chronoamperometry	Medium	30	12 ~ 6250	3	[37]
N, Cl-CDs@Van	Fluorescence	Medium	30	10^2^~10^7^	10	[38]
CCR-LFB	Colour/ Fluorescence	Low	70	10^2^~10^7^	63	[39]
Tapered SNSFC biosensor	Laser	Medium	30	70 ~ 7 × 10^4^	3.1	[40]
SC-FET/s-SWNT/RCD-Nanotree	I-V	Low	5	1 ~ 10^5^	1	This work

### Applications of SC-FET/s-SWNT/RCD-nanotree systems in real samples

To demonstrate its applicability, SC-FET/s-SWNT/RCD-Nanotree was employed to measure the real samples, including lake water, milk, and juice. Before the measurement, different samples were spiked in known quantities of SA to obtain different SA concentrations (10^2^, 10^3^, and 10^4^ CFU/mL), which were further treated with a 0.22 μm syringe filter to remove insoluble impurities. The detecting process followed the description in Sect. 2.6 to obtain the relative resistance. Table 2 shows the SA concentrations of different samples calculated using the linear regression equation in Sect. 3.4. The measured SA exhibited recoveries ranging from 91.33 to 109.41% with RSDs (*n* = 3) below 20%, indicating that the proposed method was accurate and reproducible.

**Table 2:** Applicability of SC-FET/s-SWNT/RCD-Nanotree for SA determination in different real samples

Sample	Added (CFU/mL)	Proposed method (CFU/mL)	Recovery (%)	RSD (%)
Lake water 1	5.0 × 10^2^	4.95 × 10^2^±3.72 × 10^1^	98.93	7.52
Lake water 2	5.0 × 10^3^	5.28 × 10^3^±2.92 × 10^2^	105.65	5.53
Lake water 3	5.0 × 10^4^	5.11 × 10^4^±5.84 × 10^3^	102.19	11.43
Milk 1	5.0 × 10^2^	4.74 × 10^2^±4.60 × 10^1^	94.73	9.71
Milk 2	5.0 × 10^3^	4.61 × 10^3^±3.45 × 10^2^	92.15	7.49
Milk 3	5.0 × 10^4^	5.47 × 10^4^±4.15 × 10^3^	109.41	7.58
Juice 1	5.0 × 10^2^	4.56 × 10^2^±7.27 × 10^1^	91.33	15.91
Juice 2	5.0 × 10^3^	5.12 × 10^3^±3.27 × 10^2^	102.49	6.38
Juice 3	5.0 × 10^4^	4.86 × 10^4^±6.32 × 10^3^	97.23	12.99

## Conclusions

A controllable self-cleaning FET was developed using cellulose paper, hydroxylated SWNT conductive ink, semiconducting SWNT ink and polymeric PTS nanocomposite. Then, the hydrophilicity of the biosensing areal surface was regulated using O_2_ plasma, which could significantly improve the mechanical stability, provide complex biochemical modifications, and enhance the anti-fouling performance at a low cost. By utilizing “similar DNA-origami technology”, an RCD-based DNA nanotree was first designed and then functionalized with SC-FET/s-SWNT to enhance the sensitivity of controllable self-cleaning FET biosensor for several degrees. The SC-FET/s-SWNT/RCD-Nanotree sensor could detect values as low as 1 CFU/mL within 5 min, and it had additional benefits, such as portability, user-friendliness, and no cultivation requirement. Moreover, the comprehensive performance of SC-FET/s-SWNT/RCD-Nanotree reached or even exceeded other reported sensors.

### Electronic supplementary material

Below is the link to the electronic supplementary material.

Supplementary Material 1

## Data Availability

No datasets were generated or analysed during the current study.
