# Supplementary material for: Controllable self-cleaning FET self-assembled RNA-cleaving DNAzyme based DNA nanotree for culture-free Staphylococcus aureus detection
Source: J Nanobiotechnology. 2024 Jul 15;22:414. doi: 10.1186/s12951-024-02682-3 (PMC11247881; doi:10.1186/s12951-024-02682-3)
Supplement: Supplementary file 1 — Supplementary Material 1 [file 12951_2024_2682_MOESM1_ESM.docx]

*Supporting Information*

**Controllable self-cleaning** **FET self-assembled** **RNA-cleaving DNAzyme based DNA nanotree for** **culture-free *staphylococcus aureus* detection**

Hui Wang ^a^, Ruipeng Chen ^a^, Yue He ^a,c^, Xiaoyan Zhu ^a^, Zhixue Yu ^a^, Zemeng Feng ^b^, Dongxia Pan ^a^, Liang Yang ^a^*, Xiangfang Tang ^a^*, Benhai Xiong ^a^ *

1. State Key Laboratory of Animal Nutrition and Feeding, Institute of Animal Science, Chinese Academy of Agricultural Sciences, Beijing, 100193, P. R. China.
2. Institute of Subtropical Agriculture, Chinese Academy of Sciences, Changsha, 410125, China
3. State Key Laboratory of Animal Nutrition and Feeding, College of Animal Science and Technology, China Agricultural University, Beijing, 100193, P. R. China

**Table S1.** DNA oligonucleotides in this study.

| Name | Sequence and modifications (from 5’ -terminus) |
| --- | --- |
| SA-substrate without ’rA’ | GAG AGA GAG AGA GAG TTT TCT AAT GAG TAC CTA CTG TCT CTG GAT GAT CCT ATG AAC TGA CTA TGA CCT CAC TAC CAA G |
| SA-substrate | GAG AGA GAG AGA GAG TTT TCT AAT GAG TAC CTA CTG TCT CTG GAT GAT CCT ATG AAC TGA CTrA TGA CCT CAC TAC CAA G |
| SA-DNAzyme | ATG CCA TCC TAC CAA CCA CGA AGT ACA TTT CAA ACT CAT AAC AAT CCA TCG GTT AGG TCC TGG TTG GAG CTC TGA ACT CGA GAC AGT AGG TAC TCA TTA GTT TTG AGA GAG AGA GAG AG |
| Branch-05 | CTC TCT CTC TCT CTC GTA GGA ATG GAG CTT TCG CCT GCA CTC TAC CTA GCT CCA TAT CGG ATG TTC GCC TGC ACT CTA CCT GAT CCG ATT CGT TGG ATT CGC CTG CAC TCT ACC TTC CAA CGT TCC TAC |
| Branch -03 | GTA GGA ATG GAG CTT TCG CCT GCA CTC TAC CTA GCT CCA TAT CGG ATG TTC GCC TGC ACT CTA CCT GAT CCG ATT CGT TGG ATT CGC CTG CAC TCT ACC TTC CAA CGT TCC TAC CTC TCT CTC TCT CTC |

**Table S2.** DNA oligonucleotides for RCD-Nanotree.

| Name | Sequence and modifications (from 5’ -terminus) |
| --- | --- |
| SA-DNAzyme | ATG CCA TCC TAC CAA CCA CGA AGT ACA TTT CAA ACT CAT AAC AAT CCA TCG GTT AGG TCC TGG TTG GAG CTC TGA ACT CGA GAC AGT AGG TAC TCA TTA GTT TT GCGCTATCGGGAAG |
| SA-substrate | GCGCTATCGGGAAG TTT TCT AAT GAG TAC CTA CTG TCT CTG GAT GAT CCT ATG AAC TGA CT/rA/ TGA CCT CAC TAC CAA G-(CH_2_)_6_-NH_2_ |
| YA-1 | CTTCCCGATAGCGCCCTCTGATCTAGTATGTTACTCTGCGTGCTTCCCGATAGCGC |
| YA-2 | CTTCGACGGTCATGTACTAGATCAGAGGCTTCCCGATAGCGC |
| YA-3 | CACGCAGAGTAACACATGACCGTCGAAG |
| YB-1 | CACGCAGAGTAACACATGACCGTCGAAGCTTCCCGATAGCGC |
| YB-2 | CTTCGACGGTCATGTACTAGATCAGAGGCTTCCCGATAGCGC |
| YB-3 | CCTCTGATCTAGTATGTTACTCTGCGTGCTTCCCGATAGCGC |
| YC-1 | CACGCAGAGTAACACATGACCGTCGAAGGCGCTATCGGGAAG |
| YC-2 | CTTCGACGGTCATGTACTAGATCAGAGGGCGCTATCGGGAAG |
| YC-3 | CCTCTGATCTAGTATGTTACTCTGCGTGGCGCTATCGGGAAG |
| CS-B | GCGCTATCGGGAAG |
| CS-C | CTTCCCGATAGCGC |


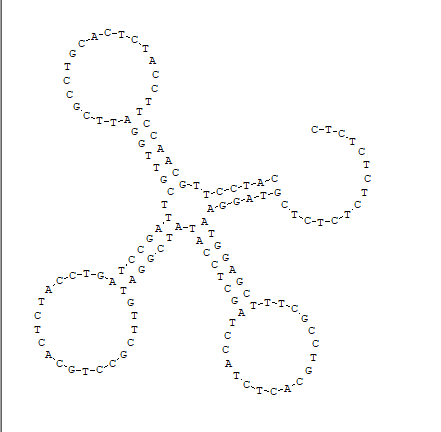

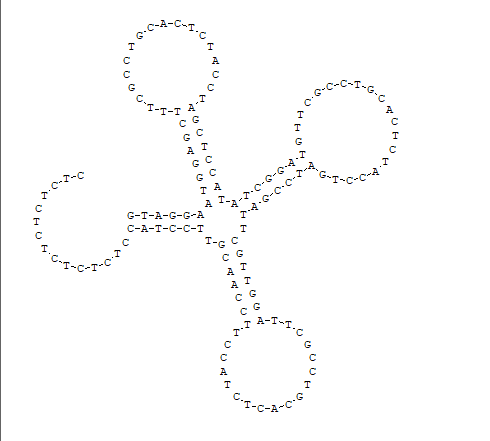


1. Branch-05 (B) Branch-03

**Figure S1.** The second structure of Branch-05 and Branch-03


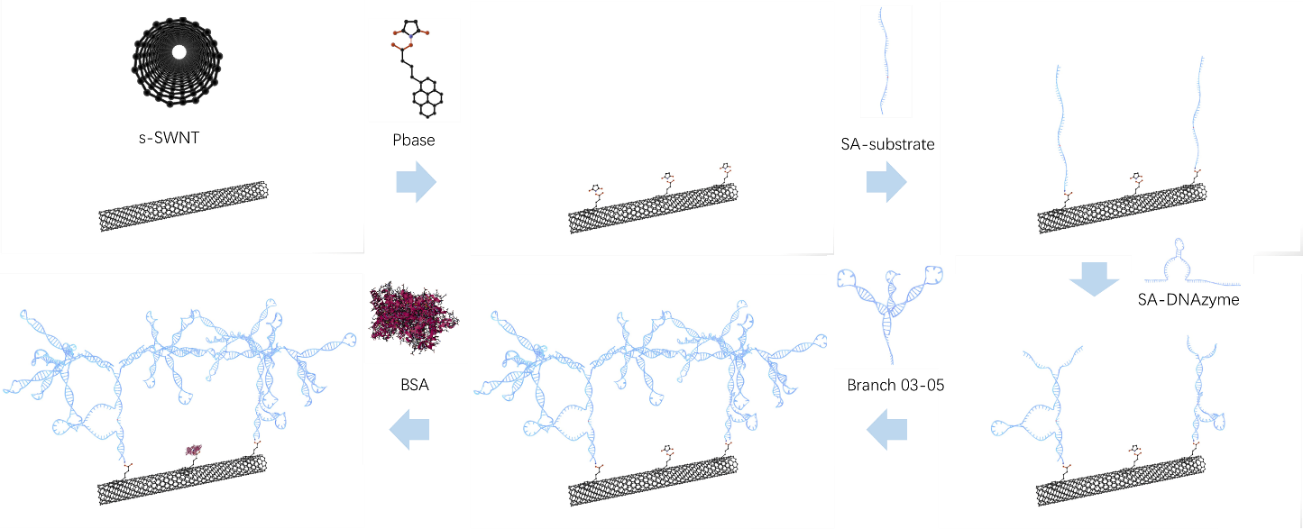


**Figure S2**. SC-FET/s-SWNT functionalized with different materials including Pbase, SA-substrate, SA-DNAzyme, Branch-03, Branch-05, and BSA


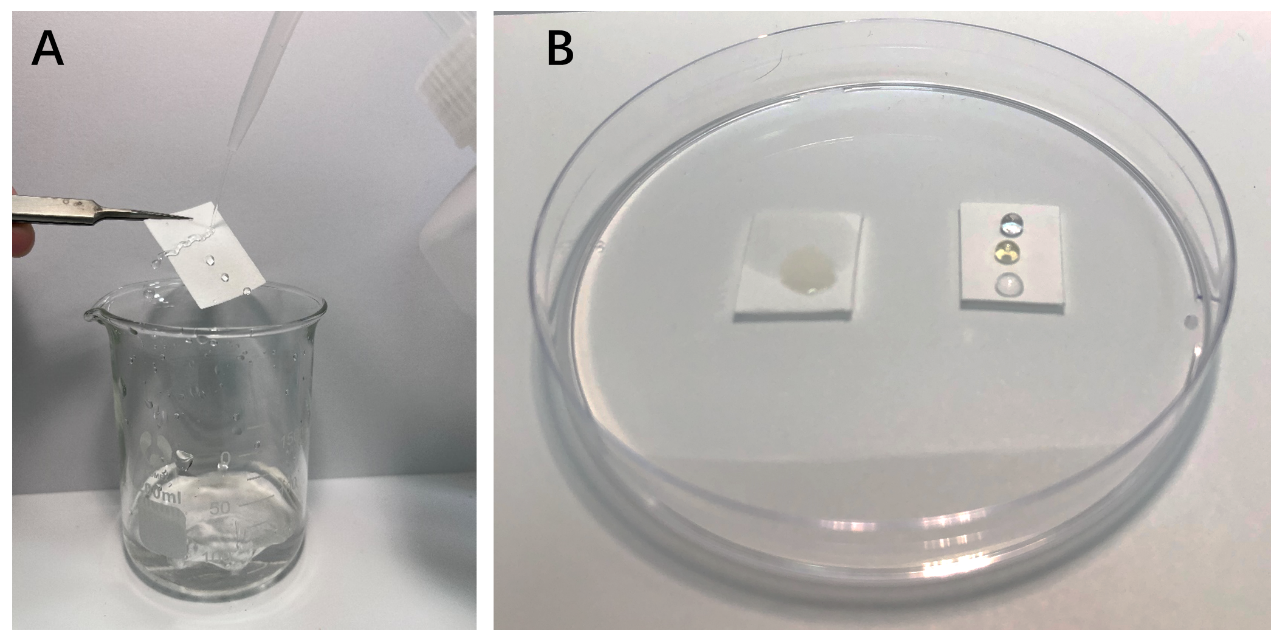


**Figure S3**. (A)Water sprayed on the surface of self-cleaning cellulose paper; (B) Different droplets of water, oil, and organic reagent on the surface of cellulose paper and self-cleaning cellulose paper.


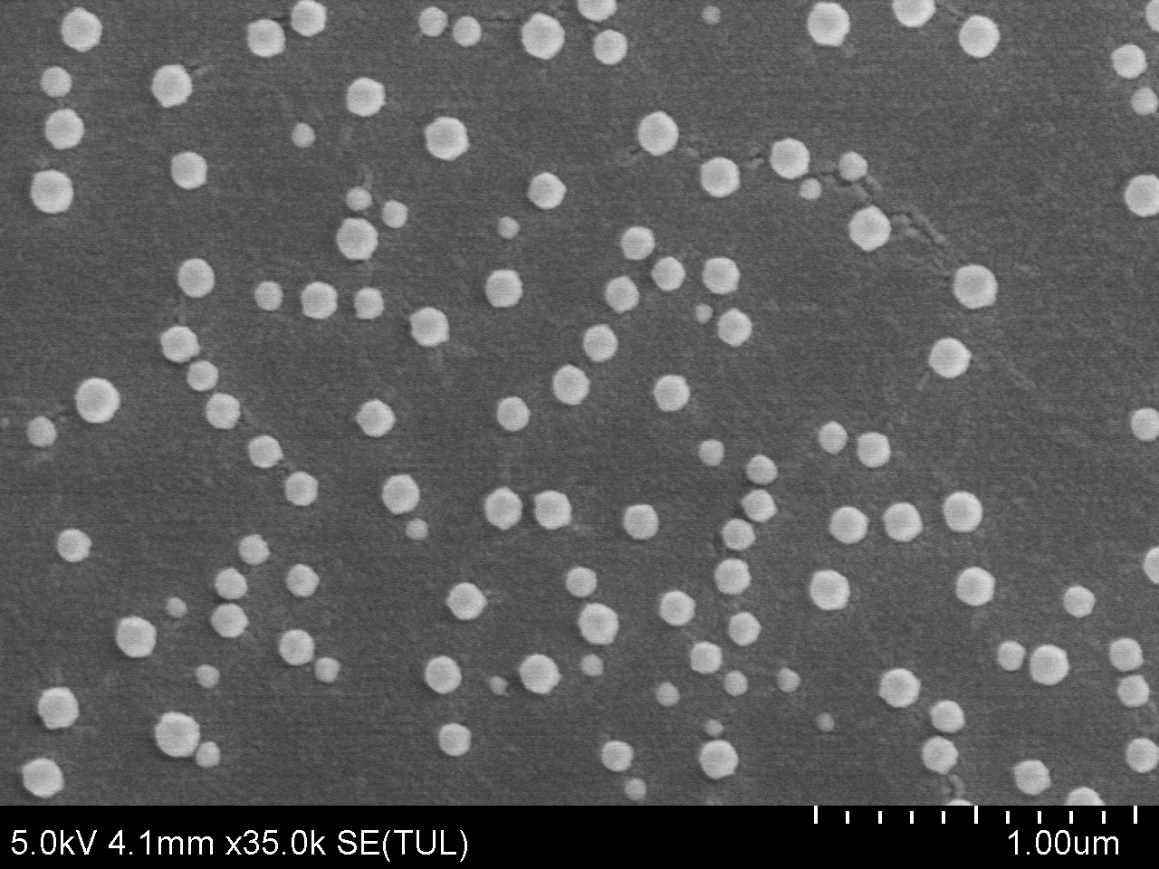


**Figure S4**. The detailed SEM of SWNT-OH based carbon nano-film functionalized with polymeric PTS nanocomposites

**Figure S5.** Resistance value at -0.1 V of SCFET/s-SWNT functionalized with Pbase, SA-substrate, SA-DNAzyme, Branch-03, Branch-05 and BSA

**Table S3.** Calculation parameters of carrier mobility

| Different modification | G_m_  (A/V) | V_th_  (V) | V_DS_  (V) | C_ox_  （F/cm^2^） | μ  (cm^2^/V·s) |
| --- | --- | --- | --- | --- | --- |
| Vs-SWNT | -1.9E-05 | -3.43 | 0.2 | 5.67E-07 | 331.76 |
| Pbase | -2.8E-05 | -2.56 | 0.2 | 1.03E-06 | 274.01 |
| SA-substrate | -4.4E-05 | -2.75 | 0.2 | 2.66E-06 | 165.26 |
| SA-DNAzyme | -7.6E-05 | -2.72 | 0.2 | 3.99E-06 | 190.19 |
| Branch | -6.6E-05 | -2.9 | 0.2 | 5.17E-06 | 127.64 |
| BSA | -0.00011 | -2.79 | 0.2 | 1.00E-05 | 111.86 |


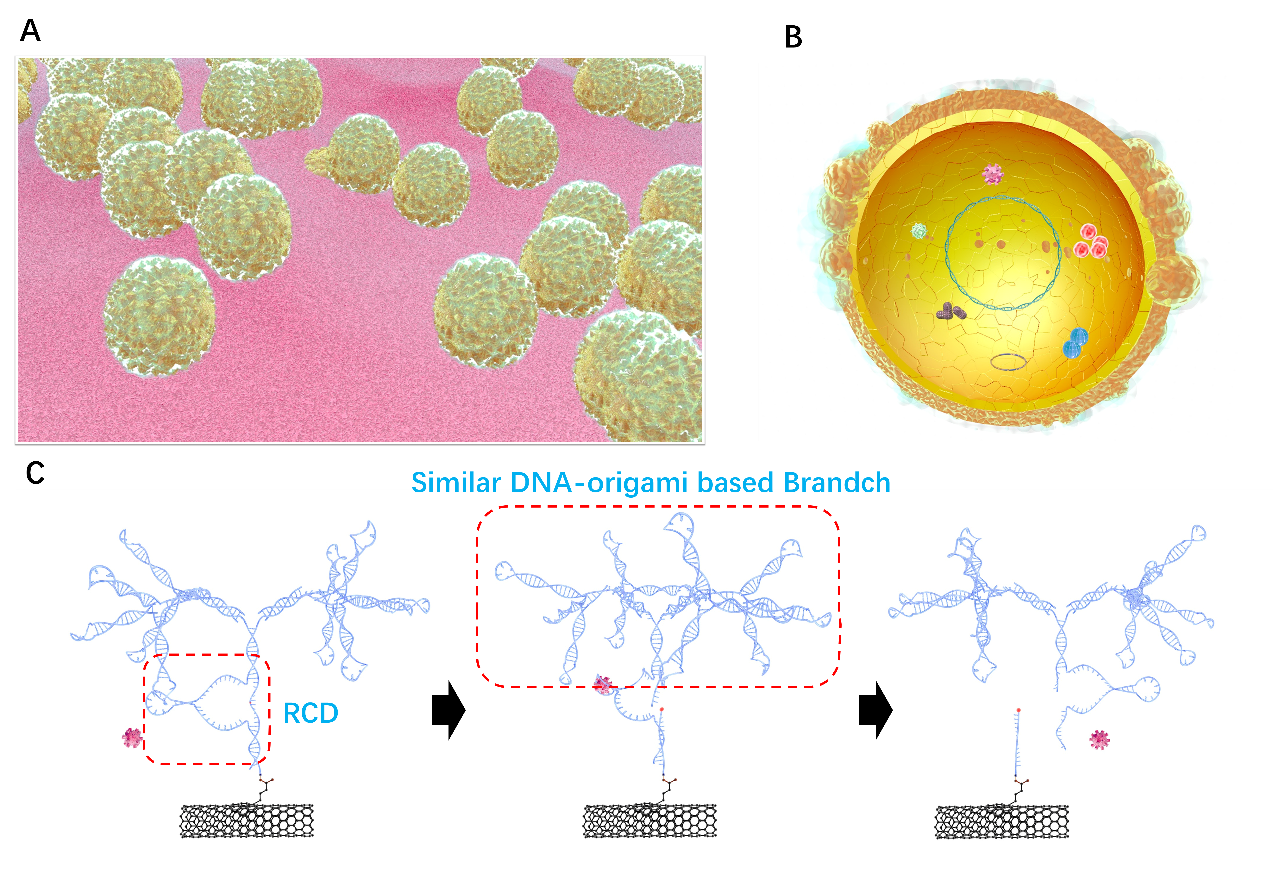


**Figure S6.** The sensitive mechanism of SC-FET/s-SWNT/RCD-Branch for SA determination

**Figure S7.** (A)Current-voltage plots, (B) Resistance-voltage and (C) Relative resistance-voltage of SC-FET/s-SWNT/RCD-Branchs to detect the different SA concentrations in the range from -0.2 V to 0.2 V with a scanning rate of 0.05 V/s


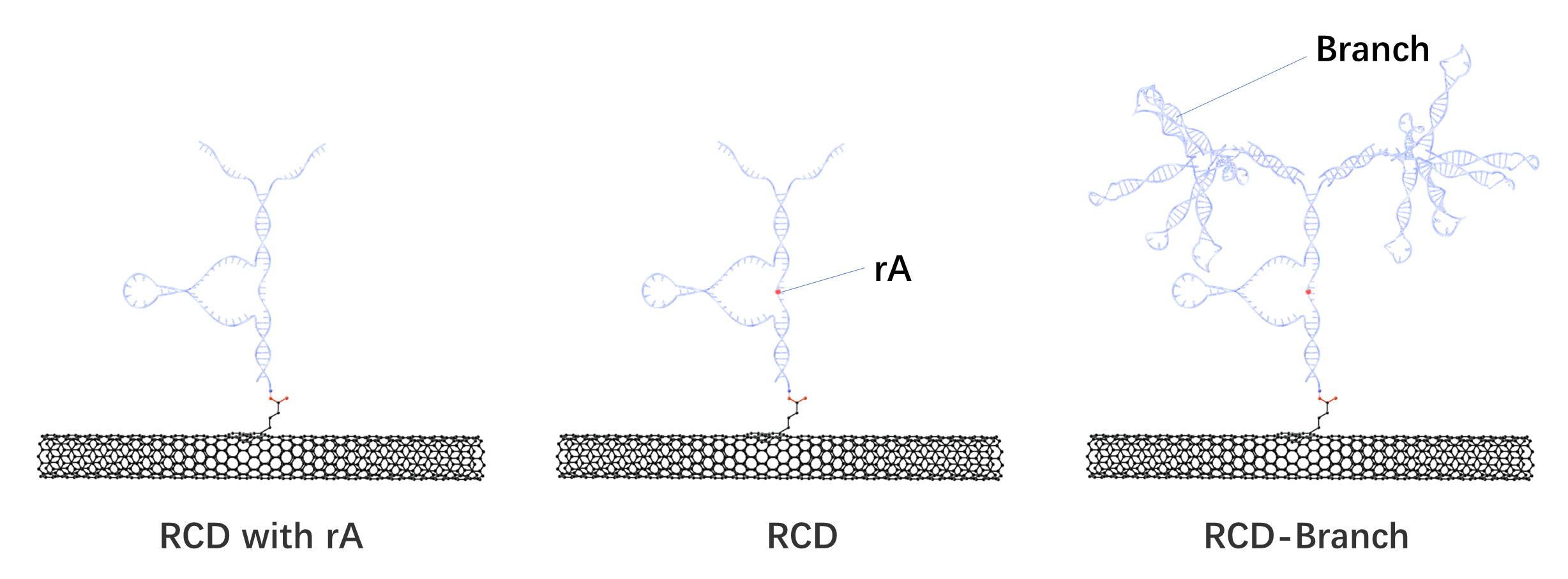


**Figure S8.** Brief diagram of different RCD-based DNA structures

**Linear relationship of SC-FET/s-SWNT/RCD-Branch**
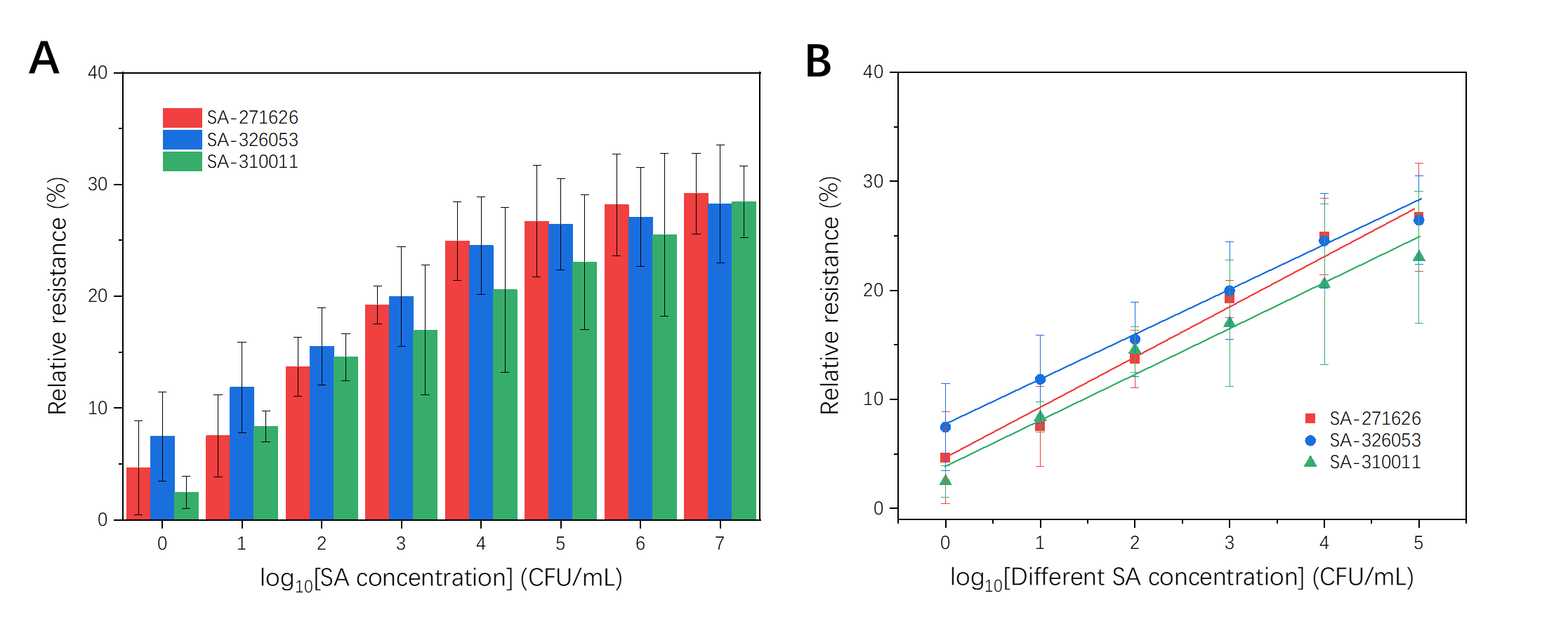


**Figure S9.** (A) Change in relative resistance after SC-FET/s-SWNT/RCD-Branch exposure to 0, 10^0^, 10^1^, 10^2^, 10^3^, 10^4^, 10^5^, 10^6,^ and 10^7^ CFU/mL of three different SA; (B) Linear regression curves and equations of three different SA detection.

The potential application of SC-FET/s-SWNT/RCD-Branch for SA detection was assessed by measuring varying concentrations of three different SA from 1 to 10^7^ CFU/mL. The results are shown in Figure 6(A). the relative resistance of the biosensor changes more rapidly at lower SA concentrations, which the calibration curve shows is a dynamic variation with the logarithm of the SA concentration in the range from 1 to 10^5^ CFU/mL. Three linear regression equations in Figure 6(B) were very close, indicating that this device has similar detection performance. This means that the biosensor can be used to roughly evaluate the number of different SA types with the same level of accuracy. The limit of detection was calculated to be 1 CFU/mL.

SA-271626:  $Relative resistance=3.9279{log}_{10}\left( SA concentration \right)+7.8044 (R^{2}=0.99)$

SA-326053: $Relative resistance=4.8031{log}_{10}\left( SA concentration \right)+4.1142 (R^{2}=0.98)$

SA-310011: $Relative resistance=4.0571{log}_{10}\left( SA concentration \right)+4.1928 (R^{2}=0.99)$
